# Energy Sources of the Depth-Generalist Mixotrophic Coral *Stylophora pistillata*

**DOI:** 10.3389/fmars.2020.566663

**Published:** 2020-11-19

**Authors:** Stephane Martinez, Yuval Kolodny, Eli Shemesh, Federica Scucchia, Reinat Nevo, Smadar Levin-Zaidman, Yossi Paltiel, Nir Keren, Dan Tchernov, Tali Mass

**Affiliations:** 1 Department of Marine Biology, The Leon H. Charney School of Marine Sciences, University of Haifa, Haifa, Israel; 2 Morris Kahn Marine Research Station, The Leon H. Charney School of Marine Sciences, University of Haifa, Sdot Yam, Israel; 3 Applied Physics Department, The Hebrew University of Jerusalem, Jerusalem, Israel; 4 The Center for Nanoscience and Nanotechnology, The Hebrew University of Jerusalem, Jerusalem, Israel; 5 The Interuniversity Institute of Marine Sciences, Eilat, Israel; 6 Department of Biomolecular Sciences, Weizmann Institute of Science, Rehovot, Israel; 7 Department of Chemical Research Support, Weizmann Institute of Science, Rehovot, Israel; 8 Department of Plant and Environmental Sciences, The Alexander Silberman Institute of Life Sciences, The Hebrew University of Jerusalem, Jerusalem, Israel

**Keywords:** mesophotic reef, photosynthesis, isotope analysis, transmission electron microscopy, photoacclimation, amino acids, transplantation

## Abstract

Energy sources of corals, ultimately sunlight and plankton availability, change dramatically from shallow to mesophotic (30–150 m) reefs. Depth-generalist corals, those that occupy both of these two distinct ecosystems, are adapted to cope with such extremely diverse conditions. In this study, we investigated the trophic strategy of the depth-generalist hermatypic coral *Stylophora pistillata* and the ability of mesophotic colonies to adapt to shallow reefs. We compared symbiont genera composition, photosynthetic traits and the holobiont trophic position and carbon sources, calculated from amino acids compound-specific stable isotope analysis (AA-CSIA), of shallow, mesophotic and translocated corals. This species harbors different Symbiodiniaceae genera at the two depths: *Cladocopium goreaui* (dominant in mesophotic colonies) and *Symbiodinium microadriaticum* (dominant in shallow colonies) with a limited change after transplantation. This allowed us to determine which traits stem from hosting different symbiont species compositions across the depth gradient. Calculation of holobiont trophic position based on amino acid δ^15^N revealed that heterotrophy represents the same portion of the total energy budget in both depths, in contrast to the dogma that predation is higher in corals growing in low light conditions. Photosynthesis is the major carbon source to corals growing at both depths, but the photosynthetic rate is higher in the shallow reef corals, implicating both higher energy consumption and higher predation rate in the shallow habitat. In the corals transplanted from deep to shallow reef, we observed extensive photo-acclimation by the Symbiodiniaceae cells, including substantial cellular morphological modifications, increased cellular chlorophyll a, lower antennae to photosystems ratios and carbon signature similar to the local shallow colonies. In contrast, non-photochemical quenching remains low and does not increase to cope with the high light regime of the shallow reef. Furthermore, host acclimation is much slower in these deep-to-shallow transplanted corals as evident from the lower trophic position and tissue density compared to the shallow-water corals, even after long-term transplantation (18 months). Our results suggest that while mesophotic reefs could serve as a potential refuge for shallow corals, the transition is complex, as even after a year and a half the acclimation is only partial.

## Introduction

Hermatypic corals are mixotrophic organisms, able to fix inorganic carbon through the activity of their dinoflagellate symbionts that belong to the family Symbiodiniaceae (Rahav et al., 1989; Muscatine, 1990; LaJeunesse et al., 2018). They are also able to gain nutrients (such as nitrogen and phosphorus) from predation of plankton (Sebens et al., 1996; Ferrier-Pagès et al., 1998; Houlbrèque et al., 2003; Palardy et al., 2006) and uptake of dissolved organic (Al-Moghrabi et al., 1993; Grover et al., 2006) or inorganic nutrients (Grover et al., 2002) from the water column. While symbionts need light for photosynthesis and constrain their host to grow mainly in the top 30 m of the ocean, corals can also form large mesophotic reefs in much deeper waters. Mesophotic coral ecosystems are reefs found deeper than 30 m where light becomes a limiting factor (Hinderstein et al., 2010). Mesophotic reefs can extend to more than 100 m depth in locations with clear water, such as the Red Sea (Lesser et al., 2009; Winters et al., 2009). Although photosynthesis is limited by the low light levels and spectral composition, these reefs host highly diverse taxa (Frederiksen et al., 2006; Leichter and Genovese, 2006). The conditions at the shallow and mesophotic reefs are markedly different. Light intensity and spectrum are the most significant varying factors, and photosynthetically available radiation (PAR) is thought to be the most limiting factor for mesophotic reefs (Kahng et al., 2019). Some coral species are endemic to only one of these ecosystems, found at strictly shallow or strictly mesophotic depths, while others are depth generalists. Such generalists, corals of the same species found along a broad depth gradient, have adapted to handle the range of conditions found in these distinct environments. In these two habitats, depth generalist corals differ in their colony morphology (Einbinder et al., 2009; Malik et al., 2020), skeletal structure (Bruno and Edmunds, 1997; Einbinder et al., 2009; Goodbody-Gringley et al., 2015; Goodbody-Gringley and Waletich, 2018; Malik et al., 2020), heterotrophic feeding, Symbiodiniaceae genera, and photosynthetic traits (Mass et al., 2007; Kahng et al., 2019).

One of the adaptations strategy of corals to mesophotic reefs relates to the ability of the symbionts to modify their photosynthetic traits. The symbiotic dinoflagellates (commonly referred to as “zooxanthellae”) belong to the family Symbiodiniaceae, comprised of several phylogenetic genera (LaJeunesse et al., 2018). The different genera exhibit varying tolerances to environmental conditions and stressors, and particularly to different light regimes (Finney et al., 2010; Eckert et al., 2020 and references therein). Zonation with depth was observed for symbionts taxa in some cases (Rowan and Knowlton, 1995; Winters et al., 2009; Borell et al., 2016), and these differences were even suggested to be responsible for the vertical distribution of coral species in some places (Iglesias-Prieto et al., 2004). Still, members of all genera use photo-acclimation strategies when exposed to different light intensities (Chang et al., 1983; Kaiser et al., 1993). However, these photo-physiological responses differ between species and opposing photo-acclimatization has been reported even for the same species at varying conditions (Kahng et al., 2019).

The second adaptation strategy is related to the host and its predation capabilities. Heterotrophy has been suggested not to represent a significant proportion of the total energy budget of corals living in shallow waters, and to increase its part in corals inhabiting deep or turbid waters, i.e., low-light environments (Muscatine and Kaplan, 1994; Anthony and Fabricius, 2000). The logic behind this concept is the assumption that the total energy budget should be maintained. Therefore, in places with high light, corals allegedly rely primarily on their symbionts for fixed carbon whereas at low light they switch to their alternate source of energy, i.e., predation (Muscatine et al., 1989). Hence, it is expected that corals that live at mesophotic depths will have a higher trophic position (TP) than those that live in shallow water. Other observations, however, tend to support the idea that heterotrophy can be just as important at all depths and light environments (Muscatine and Kaplan, 1994; Houlbrèque et al., 2003; Palardy et al., 2006). While most ways to test and calculate the TP in ecology do not apply to corals (e.g., stomach content), tissue stable isotope analysis might be applicable. The classic approach is through analysis of bulk tissue nitrogen and posits that δ^15^N increases (become enriched) with higher TP since each consumer will be enriched relative to its prey (Minagawa and Wada, 1984). Therefore, the results are always relative to another organism and do not provide an absolute TP number, as δ^15^N is not constant under all conditions but is instead influenced by food sources, stress, consumer physiology, and the background δ^15^N of the surrounding environment (Popp et al., 2007). Constraining the nitrogen isotopic baseline, or isotopic composition of primary producers at the base of an ecosystem can be complicated and may be difficult or impossible in many environments (Popp et al., 2007). This problem seems to be significant in corals, where anticipated differences between corals with predicted differing TPs are not evident or are hard to separate from other forces that change δ^15^N (Houlbrèque and Ferrier-Pagès, 2009; Nahon et al., 2013; Fox et al., 2019; Wall et al., 2020).

McClelland and Montoya (2002) were the first to examine the amino acids compound-specific stable isotope (AA-CSIA) in the relationship between phytoplankton and their consumer zooplankton in a controlled laboratory setting. They discovered that the non-essential amino acid (AA) glutamic-acid (also known as “trophic” AA) is enriched in δ^15^N compared to the bulk tissue. The essential AA phenylalanine (also known as “source” AA) is not affected by the organisms’ TP and does not become enriched relative to an organism’s prey. Therefore, this source AA “records” the δ^15^N of the primary producers in the particular food web in question. Since both isotopic baseline and fractionation information is retained in the sample that enables the calculation of the trophic position from a single consumer independently of the surrounding values. Later studies tested several different macroalgae, phytoplankton, zooplankton gastropods, and fish in the natural environment and lab (Chikaraishi et al., 2007, 2009). It was concluded that due to the different traits of the AAs (glutamic-acid and phenylalanine), they can give an indication of the TP (Chikaraishi et al., 2010). Since AA contain carbon atoms as well as nitrogen, it is possible to extend the isotopic analysis even further to trace the different carbon sources between shallow and mesophotic reefs using the carbon analysis in AA-CSIA. Essential AAs δ^13^C values are representing the isotopic signature of primary producers at the base of a food web without the confounding influence of trophic fractionation (Edgar Hare et al., 1991; Johnson et al., 1998; Howland et al., 2003; McMahon et al., 2010). Compared to conventional bulk stable isotope analysis (SIA), AA-CSIA δ^13^C analysis is a more powerful tool for examining changes in diet and habitat (McMahon et al., 2010, 2011, 2012, 2015), carbon source (McMahon et al., 2010), and links to a specific habitat (McMahon et al., 2012). Combining carbon and nitrogen AA-CSIA will enhance the understanding of the food webs of coral reefs found at various depths.

Since, mesophotic reefs have not experienced the recent coral decline trends of their shallow-water counterparts, displaying relatively stable coral populations over time (Bak et al., 2005). It is hypothesized that mesophotic coral reefs may serve as an important refuge for coral species, thereby increasing overall reef resilience (Glynn, 1996; Bongaerts et al., 2010). The resilience of ecosystems under rapid environmental change relies, in part, on the capacity of organisms to adapt and/or acclimatize to new conditions. Settling larvae from mesophotic reefs capture the highest potential to colonize the shallow reef, however studying the acclimation mechanisms of mature coral transplants is beneficial for several reasons. The survival of larvae in a shallow reef depends on both its intrinsic acclimation capability and its ability to recruit suitable symbionts or its symbionts to acclimate to the higher light regime. Transplantation of coral fragments provides the opportunity to distinguish between acclimation processes that depend on the symbionts and these that depend on the host. Studying long-term transplantation from mesophotic to shallow reefs is required to inform restoration projects, in which damaged habitats are re-introduced with coral fragments from mesophotic reefs (Abelson, 2006; Tortolero-Langarica et al., 2014). It was also suggested as a way to promote attraction and seeding of larvae (Ferse et al., 2013).

In this study, we examined the hermatypic coral *Stylophora pistillata,* a depth-generalist, in the Gulf of Aqaba in order to determine its energy sources in shallow and mesophotic reef and their adaptation and/or acclimatization from mesophotic to shallow reefs that will ultimately indicate the potential of mesophotic reefs to serve as refuges. In this region, S. *pistillata* has a well-defined vertical zonation of Symbiodiniaceae genera: deep-water colonies (>20 m) host symbionts belonging to *Cladocopium goreaui* (former clade C typical for low light habitats), whereas colonies growing in shallow water (<20 m) host *Symbiodinium microadriaticum* (former clade A typical for high light habitats) (Winters et al., 2009; Borell et al., 2016). Hence, a simple comparison between shallow and mesophotic *S. pistillata* colonies is a comparison between the same host species, but with a different symbiont. Therefore, it is not possible to attribute observed differences in holobiont physiology to the adaptation of either the host or the symbiont, as they may simply arise from the different symbiont genera (Wall et al., 2020). In the present work, stepwise transplantation of *S. pistillata* from 60 to 5 m reefs was conducted. The transplants were sampled after 1δ months and compared to shallow and mesophotic colonies in their photosynthesis characteristics, trophic position, and carbon source using diverse physiological tools, transmission electron microscopy, and AA-CSIA. This transplantation is used as a methodology to overcome the limitation of different Symbiodiniaceae genera as well as examining the long-term adaptation of the transplants to the new environment.

## Materials And Methods

### Sample Collection

Colonies of the hermatypic coral *S. pistillata* (Esper, 1797) were collected under a special permit by the Israel Nature and Parks Authority from depths of 5 and 60 m in front of the Interuniversity Institute for Marine Science (IUI) at the Gulf of Eilat, in the northern Red Sea (29º 30’ N, 34º 56’ E). Dives were accomplished using Megalodon closed-circuit rebreathers (Inner space Systems) as well as NITROX SCUBA.

### Transplantation

We transplanted five coral colonies from 60 to 5 m following the protocol described by Cohen and Dubinsky (2015). Briefly, all five coral colonies from 60 m were fragmented, the fragments were fixed onto a table (1 m × 2 m), and over the course of 3 months and six stations, they were gradually moved to their final destination in the 5 m reef. Eighteen months following the completed transplantation from mesophotic to shallow, three fragments were sampled from the transplanted colony and three random colonies in the 5 m reef (shallow) and 60 m reef (mesophotic) at the same locations were the transplantation took place. The samples were analyzed for alga population, pigment content, photosynthetic performance, and AA-CSIA signature.

### Host Protein, Symbiodiniaceae Isolation and Chlorophyll Analysis

Fragments were flash-frozen in liquid nitrogen and kept at –80ºC. Coral tissue was removed by an airbrush connected to a reservoir of phosphate buffer saline (PBS) solution filtered through a 0.22 μm filter; skeletons were then kept for further analysis. The extracted tissue was mechanically disrupted using an electrical homogenizer (HOG-160-1/2, MRC-labs, Israel) for 3 s × 10 s.

The homogenate was centrifuged at 5000 *g* for 5 min at 4°C to separate the debris and the symbiont cells from the coral host tissue. A protease inhibitor cocktail (cat. G652A, Promega, United States) was added to the supernatant with the host tissue and sonicated for 3 s × 30 s (Ultrasonic cell crusher, MRC-labs, Israel). Coral host total protein concentration was quantified using a photometric BCA protein kit (Pierce BCA, United States) following the manufacturer’s protocol on a Perkin Elmer plate reader (2300 EnSpire^®^, United States) at 540 nm emission.

Symbiont concentration of the homogenate was determined by fluorescent microscope counts (Nikon Eclipse Ti, Japan) using a hemocytometer (BOECO, Germany) with 5 replicate (1 mm^2^each) cell counts per sample. Each replicate was photographed both in brightfield and in fluorescent light 440 nm emission to identify chlorophyll. Cell counting was performed using NIS-Elements Advanced Research (version 4.50.00, Nikon, Japan) with 0.5 < Circularity < 1 and the typical diameter parameter was to set between 5 to 15 μm.

Chlorophyll concentrations were measured on 2 ml of tissue homogenate that was filtered onto a Whatman GF/C filter and incubated overnight with 1 ml of 90% cold acetone at 4°C. After incubation, the filter was manually homogenized, and the solution was filtered into a glass cuvette through a 0.22 μm syringe filter. A NanoDrop (Thermo Fisher Scientific, United States) was used for photometric measurements at wavelengths of 630, 647, 664, and 691 nm, and chlorophyll *a* and *c* concentrations were calculated according to the equations of Ritchie (2008).

### Photophysiology

Following retrieval, all fragments were immediately dark incubated for 4 h in ambient seawater, while taken to the laboratory. Three fragments from each colony were measured using Imaging-PAM (Maxi-version, Walz GmbH, Effeltrich, Germany), to determine maximal photochemical efficiency of dark-adapted RCIIs *(Fv/Fm)* values, and to obtain light-response curves of apparent effective photochemical efficiency of RCIIs in actinic light (YII) and non-photochemical quenching (NPQ). Extracted symbionts’ fluorescence spectra were measured using a Horiba PTI Quantamaster (Kyoto, Japan) fluorometer.

### Symbiodiniaceae ITS2 Analyses

Genomic DNA was extracted from the three colonies of each treatment (shallow, deep and transplanted, nine samples in total) using the Wizard genomic DNA purification kit (Promega Corporation, United States). The internal transcribed spacer (ITS2) region of Symbiodiniaceae rDNA was amplified using Symbiodiniaceae-specific primers adapted from Arif et al. (2014) to which an Illumina CS1 tag added (underline sequence): Forward ACA CTG ACG ACA TGG TTC TAC ATG TGA ATT GCA GAA CTC CGT G Reverse: TAC GGT AGC AGA GAC TTG GTC TTA CTT ATA TGC TTA AAT TCR GCG G. The PCR products were sent to HyLab (Hy Laboratories Ltd., Israel) where they were subjected to a second PCR using the Access Array tag for Illumina primers (Fluidigm Corporation, United States). This second PCR added the index and adaptor sequences required for sequencing on the Illumina system. The samples were then purified using AMPure XP beads (Beckman Coulter Inc., United States) and the concentration was determined by Qubit (Thermo Fisher Scientific, United States). The samples were pooled together and sequenced on the Illumina MiSeq using a v2-500 cycle kit to generate 250 × 2, paired-end reads. The data were de-multiplexed by the Illumina software, and the de-multiplexed FASTQ files were further analyzed. The resulting operational taxonomic units (OTUs) were aligned with ClustalX (Larkin et al., 2007) and blasted in GenBank^1^.

### Transmission Electron Microscopy

To isolate the living symbionts from the host cell, two coral fragments from each treatment were incubated for 20 min in calcium-free seawater followed by disrupting the polyps by scraping the coenosarc with a 10 μl pipette tip to dislodge cells and tissue. The homogenate was centrifuged at 500 *g* for 10 min at room temperature to separate the symbiont cells from the coral host tissue. The symbionts were resuspended in 1 ml of fresh fixative buffer containing 4% paraformaldehyde and 2% glutaraldehyde in an artificial seawater solution for 2 h at room temperature followed by overnight incubation at 4ºC. The samples were rinsed twice with artificial seawater followed by centrifugation at 2000 *g* for 5 min. The cells were then post-fixed with 1% osmium tetroxide supplemented with 0.5% potassium hexacyanoferrate trihydrate and potassium dichromate in artificial seawater (1 h), stained with 2% uranyl acetate in water (1 h), dehydrated in graded ethanol solutions and embedded in Agar 100 epoxy resin (Agar Scientific Ltd., Stansted, United Kingdom). Ultra-thin sections (70–90 nm) were viewed and ∼70 chloroplasts of each treatment were photographed with an FEI Tecnai SPIRIT (FEI, Eindhoven, Netherlands) transmission electron microscope operated at 120 kV and equipped with a One View Gatan Camera. To calculate the ratio of the area occupied by membranes within a region of interest, membranes were segmented using pixel-based classification with Ilastik machine learning software (Berg et al., 2019); subsequently, the ratio was calculated using the Fiji image processing package (Schindelin et al., 2012). The analysis was performed on 40–75 chloroplasts from each group.

### Compound-Specific Stable Isotope Analysis

Coral tissue was removed using an airbrush connected to a reservoir of double-distilled water. The extracted tissue was mechanically disrupted using an electrical homogenizer (HOG-160-1/2, MRC-labs, Israel) for 3 s × 10 s. The homogenate was centrifuged at 500 *g* for 10 min to separate the symbiont cells from the coral host tissue. The supernatant (coral tissue homogenate) was centrifuged again for 5 min at 20000 *g.* The pellet of the first centrifugation (consisting mostly of symbiont algae) was washed with double distilled water, resuspended in 1 ml double distilled water and then re-centrifuged for 5 min at 20000 *g*. Both algal cells and coral host tissue samples were lyophilized for 24 h prior to isotopic analysis.

Approximately 3 mg of lyophilized host or algal cell pellet was acid hydrolyzed in 0.5 ml of 6 nM HCl at 150°C for 75 min (Cowie and Hedges, 1992) under nitrogen atmosphere inside a 4 ml glass vial with PTFE cap. Samples were cooled to room temperature and then HCl was evaporated under a gentle stream of nitrogen. Samples were neutralized twice with 0.5 ml ultra-pure water and evaporated with a gentle stream of nitrogen. We used the EZfaast amino acid analysis kit (Phenomenex) with a slight modification of replacing reagent 6 with dichloromethane as a solvent. For carbon analysis, we injected 2 μl into a Thermo Scientific Trace 1300 gas chromatograph in split mode (1:15) at 250°C; for nitrogen, we injected 2 μl in split mode (1:5) at 250°C. Helium was used as the carrier gas at a constant flow of 1.5 ml/min. The amino acids were separated on a Zebron ZB-50 column (30 m, 0.25 mm, and 0.25 μm) with the following settings to optimize peak separation for the desired amino acids: Initial temperature 110°C ramped to 240 at 8°C per min and then ramped to 320 at 20°C per min and held for 2.5 min. The separated amino acids were split in a microchannel device into two directions, one toward a Thermo Scientific ISQ quadrupole for amino acid identification and the second toward a Thermo Scientific Delta V Advantage isotope ratio mass spectrometer for carbon and nitrogen isotope analysis. The ISQ condition was set for: transfer line 310°C, ion source 240°C and scan range from 43 to 450 m/z mass range. To define the isotopic ratio of carbon and nitrogen the separated amino acids were combusted in a Thermo Scientific GC Isolink II at 1000°C for CO_2_ and N_2_; N_2_ went through a liquid nitrogen cold trap to freeze down all other gases before entering the Delta-V. Each sample was analyzed in duplicate for carbon and triplicate for nitrogen.

### Isotope Data Analysis and Corrections

Stable isotope ratios expressed in δ notation were calculated against Vienna PeeDee Belemnite (VPDB) for carbon and atmospheric N_2_ (Air) for nitrogen. Individual AAs (Sigma Aldrich) were analyzed at the Geological Survey of Israel by elemental analyzer (1112 Flash EA, Thermo) interfaced with isotope ratio mass spectrometer (IRMS, Delta V Plus, Thermo). To extend the nitrogen isotopic range, two certified AAs (alanine + 43.25‰ and valine + 30.19‰ Arndt Schimmelmann, Biogeochemical Laboratories, Indiana University) were added. We used a standard containing seven AAs with known nitrogen isotope ratios (alanine, valine, leucine, isoleucine, methionine, glutamic acid, and phenylalanine) with nitrogen isotopic range of −6.69 to + 43.25‰ Since nitrogen is not added in the process of derivatization, corrections for nitrogen addition were not required. To account for the carbons that are incorporated during the derivatization process we determined the correction factor for each amino acid using the equation: *ncdδ13Ccd* = ncδ13Cc + *ndδ13Cdcorr* where n is the number of moles of carbon, Cc the compound of interest (here, each AA), Ccd the derivatized compound, and Cdcorr the empirically determined correction factor (Docherty et al., 2001). The standard for each AA was used to set Cdcorr before each sample’s isotope ratio calculation. The AA’s standard was injected three times after the carbon combustion reactor oxidation and three more times for nitrogen to allow for drift correction, and again three times for carbon and nitrogen after a maximum of 18 sample injections. Since AAs differ in the presence of heteroatoms and functional groups it may lead to different combustion efficiencies and therefore differences in drift, an average of the standard injection from the beginning and the end of the sequence was used. For each sequence of nitrogen, a correction factor was applied based on the linear regression equation of the ratio between the known AA isotopic ratio and the acquired result for the sequence. The trophic position was calculated from the equation TP(*Glu/Phe*) = ((δ^15^ *NGlu* − δ^15^ *NPhe* − β)/*TDF*_*AA*_) + 1 (Chikaraishi et al., 2009) where β × −0.36 and TDF_AA_ = 4.54 (Martinez et al., 2020).

### Statistical Analysis

Data on the physiological parameters were tested for normality (Shapiro–Wilk test) and homogeneity of variance (Brown-Forsythe test). One-Way ANOVA was used for all measurements, followed by Tukey test for *post hoc* comparisons, in which significant groups have a value of *p* ≤ 0.05. Non-parametric equivalents of tests were used in cases where assumptions were violated. For such cases, a Mann-Whitney test or a Kruskal-Wallis test was used, followed by Dunn’s test for multiple comparisons. The GraphPad Prism software version 8.0.2 (GraphPad Inc.) was used to perform all the statistical tests.

Statistical analysis of isotopic data was done using R v3.6.2 (R Development Core Team, 2015) and tested using permutational multivariate analysis of variance (PERMANOVA) using distance matrices test (Anderson, 2006) implemented as adonis in the Vegan package. In Adonis, Euclidean distance matrix measurements were used as a response variable, considering the additive effect of the treatment (mesophotic, shallow and translocated) and species (host and symbiont) factors (Martinez Arbizu, 2019). All *p*-values were adjusted using the Benjamini Hochberg (BH) correction factor. Only values with *p*-values or adjusted p-values < 0.05 were considered significant.

## Results

### Tissue and Algal Density

**Figure 1:**
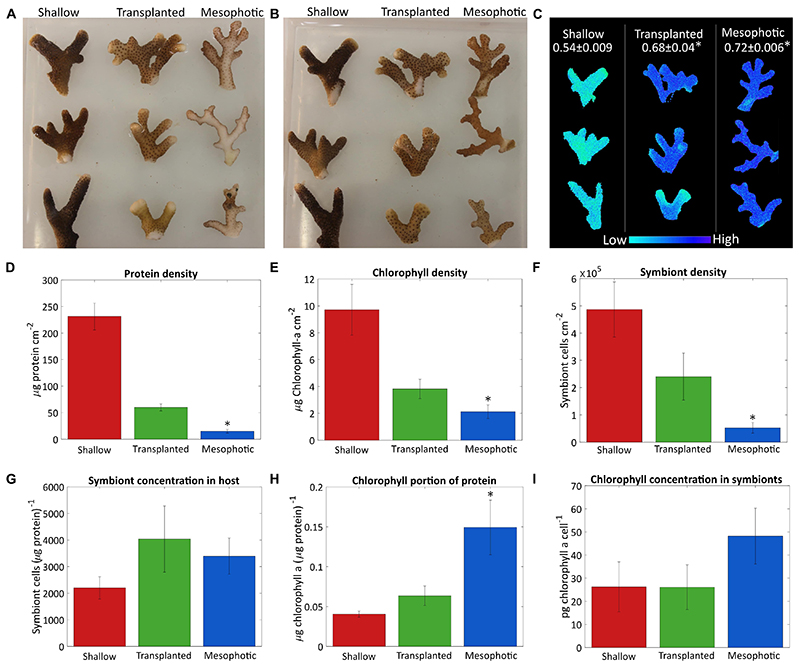
Algae coverage and chlorophyll content. Three fragments from each colony were sampled after 18 months from the shallow corals (red), transplants from mesophotic to shallow (green), and mesophotic corals (blue). Photos of (**A**) the upper side of the fragments which faces the water surface and (**B**) the bottom side of the fragments, which faces the seabed. (**C**) Maximal photosystem II quantum yield (*F_v_/F_m_*) obtained by Imaging-PAM, measured on the upper side of the fragments. Average values for each colony are given at the top. (**D**) Total protein per surface area. (**E**) Surface density of chlorophyll *a*. (**F**) Number of symbiont cells per area. (**G**) Number of symbiont cells per total protein. (**H**) Chlorophyll *a* ratio to total protein. (**I**) Cellular chlorophyll *a* concentration in the symbiont cells. Error bars of (**D-I**) showing the standard error of the mean, *n* = 4. Asterisks (*) indicate statistical differences relative to shallow colonies.

The differences observed in coral tissue, symbiont coverage and chlorophyll *a* content of the symbionts are depicted in Figure 1. Photos of the fragments from both sides (Figures 1A,B) show qualitatively that fragments from the 60 m corals colony had very low algae density on the side which faced the seabed, while the algae were distributed equally on both sides of the shallow-water coral branches. The mesophotic colony structure is flat (Einbinder et al., 2009), and the side that faces the seabed does not receive enough reflected light from the sediment, thus photosynthesis mostly takes place on the upper side of the fragments. After 18 months the deep-to-shallow transplanted fragments had increased their algal coverage so that algal density was similar on both sides and the bottom and upper sides could not be distinguished by eye anymore. However, the shallow fragments still had a noticeably darker color than the transplants, most likely due to the higher chlorophyll *a* surface density (Figure 1E). The chlorophyll *a* surface density was significantly different between the sample groups (Kruskal-Wallis rank sum test, *p* + 0.0024, *n* = 4 per group). In particular, the chlorophyll *a* surface density was significantly lower in the mesophotic corals compared to the shallow (Dunn’s test, *p* = 0.013). In the transplanted corals, the chlorophyll *a* surface density was similar to but higher than that of the mesophotic corals, reflecting the differences in color observed by eye (Figures 1A,B).

Quantitative comparison based on total protein, symbiont cell count, chlorophyll *a* extraction and surface area is presented in Figures 1D-I. Total protein and symbiont cell surface density were significantly different between corals in shallow, mesophotic, and transplanted colonies (Kruskal-Wallis rank sum test, *p* = 0.0002, 0.002, respectively, *n* = 4 per group). The shallow colonies had much higher total protein and symbiont cell surface density than those in the mesophotic colonies (Dunn’s test, *p* = 0.0051 and 0.032, respectively) (Figures 1D,F). However, the ratio of number of symbiont cells to total protein was not significantly different between the shallow and mesophotic colonies (Kruskal-Wallis rank sum test, *p* = 0.815, *n* = 4 per group, Figure 1G), indicating the number of symbionts per coral tissue is similar. Even though the mesophotic corals had overall lower chlorophyll *a* surface density compared to the shallow (Dunn’s test, *p* = 0.013) (Figure 1E), relative to the amount of tissue (Figure 1H) they had much more chlorophyll *a* (Tukey’s test, *p* = 0.028). However, although the chlorophyll *a* content of each symbiont in the mesophotic colonies is higher, this trend was not significant (Dunn’s test, *p* = 0.84, Figure 1I).

While the transplanted corals showed signs of acclimation to the shallow environment after 18 months, they clearly still resemble colonies from their original habitat, the mesophotic colonies. The chlorophyll *a* content of their symbionts decreased but was not significantly different than the shallow corals (Kruskal-Wallis rank sum test, *p* = 0.436, *n* = 4 per group, Figure 1I). Further, although the overall chlorophyll *a* portion of the total protein in the transplanted corals decreased compared to the mesophotic colonies and resembled the ratio in the shallow corals by the end of the experiment it is not significantly different than the shallow or the mesophotic colonies (Tukey’s test,*p* = 0.78 and 0.08, respectively, Figure 1H). Similar trend was observed for the rest of the parameters shown in Figures 1D-F, the transplanted corals were far from completing the shift to resemble a shallow coral. The total protein surface density increased but is still more similar to that of the mesophotic colonies. The chlorophyll *a* and symbiont surface density both increased, but were intermediate to the shallow and mesophotic levels. The ratios of symbiont cells to protein were not significantly different between the three groups (Kruskal-Wallis rank sum test, *p* = 0.815, *n* = 4 per group, Figure 1G).

### Symbionts and Photobiology

Shallow, mesophotic, and transplanted fragments exhibited significantly different *F_v_/F_m_* values (One-Way ANOVA, *p* = 0.0006, *n* = 3 per group, Figure 1C). The *F_v_/F_m_* of the mesophotic fragments (0.72 ± 0.006) was significantly higher compared to the shallow fragments (0.54 ± 0.009) (Tukey’s test, *p* = 0.0006), meaning they have a higher dynamic range in which they can utilize the absorbed energy. Translocated fragment *F_v_/F_m_* was significantly higher compared to the shallow (Tukey’s test, *p* = 0.003), and it was nearly as high as that of the mesophotic corals (0.68 ± 0.04), indicating that even after 18 months they preserved the maximal photochemical potential they had when in the mesophotic colony.

**Figure 2:**
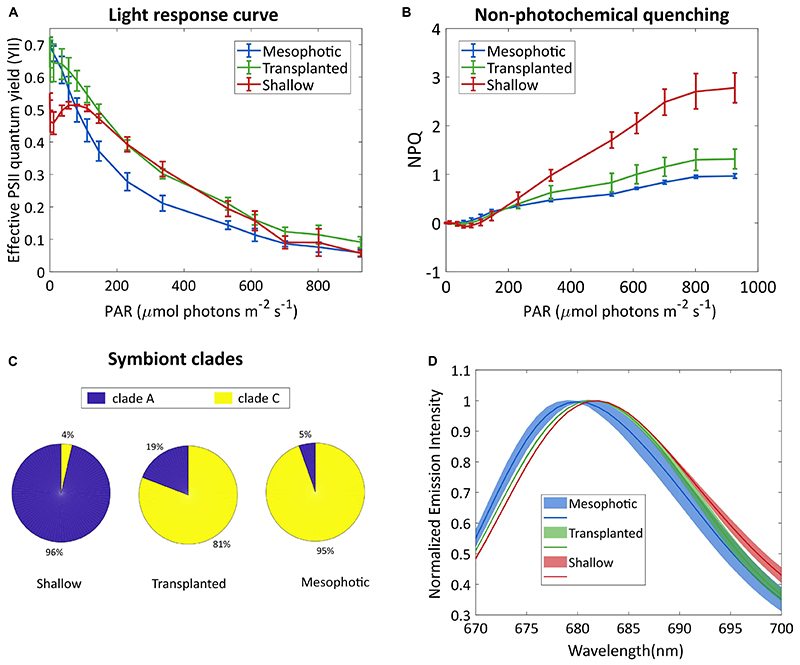
Symbionts analysis. (**A**) Effective photosystem II quantum yield of the symbionts and (**B**) non-photochemical quenching, under different irradiances. Error bars show SD of three different fragments. (**C**) Symbiont clade composition 18 months after the reciprocal transplant. SD values are ±1, ±7, and ±1.7% for the shallow, transplanted and mesophotic groups, respectively. (**D**) Fluorescence spectra of the symbionts, following excitation at 475 nm. Lines show averaged spectra, the shaded area represents the SD (*n* = 3).

To better understand the photo-acclimation process that the transplanted corals underwent, we compared their photosynthetic characteristics under different light intensities. The light response curve seen in Figure 2A shows an intriguing phenomenon. The apparent effective photochemical efficiency of RCIIs in actinic light (YII) of the symbionts in the transplanted corals is high under low light, similar to the mesophotic corals which are adapted to low light whereas the YII of the shallow colonies was lower under low light. In the region of 200-500 μmol photons *m^−2^s^−1^* the transplanted fragments performed similarly to the shallow ones, while the mesophotic symbionts performed lower. At higher light intensities (above 700 μmol photons *m^−2^s^−1^*), YII of all three groups was equally low, likely due to photoinhibition. Moreover, non-photochemical quenching (NPQ) of the mesophotic and transplanted fragments was similar and lower than that of the shallow colonies (Figure 2B).

To understand the origin of this impressive flexibility, we further probed the changes that occurred to the symbionts by determining their species and cellular structure. A total of 22,344 high-quality ITS2 sequence reads (mean length = 320 bp) were obtained from 9 total samples (three samples each from the shallow, mesophotic and transplanted fragments). A large proportion of the filtered OTUs were non-Symbiodiniaceae organisms that were likely prevalent in the water and as part of the coral holobiont. After the removal of non-Symbiodiniaceae sequences and of singletons (OTUs with 1 sequence read), OTUs clustered into *S. microadriaticum* and *C. goreaui* (former clades A and C, respectively) at 97% similarity. Mesophotic colonies contained symbiont consortia dominated by *C. goreaui* (94.6%) with a small percentage representing *S. microadriaticum* (5.4%; Figure 2C). In contrast, the symbiont consortia present in shallow colonies were dominated by *S. microadriaticum* (96.4%) with a small percentage of *C. goreaui* (3.6%) present. In the transplanted fragments only a limited change was observed in the symbiont genera even after 18 months after which less than 20% of their symbiont composition had changed from *C. goreaui* to *S. microadriaticum*.

We compared the chlorophyll fluorescence spectrum of the symbionts extracted from the different groups (Figure 2D). The fluorescence peak of the shallow corals was red-shifted compared to that of the mesophotic corals. The transplanted fragments’ symbionts fluorescence was shifted to match the spectrum of the shallow symbionts.

**Figure 3:**
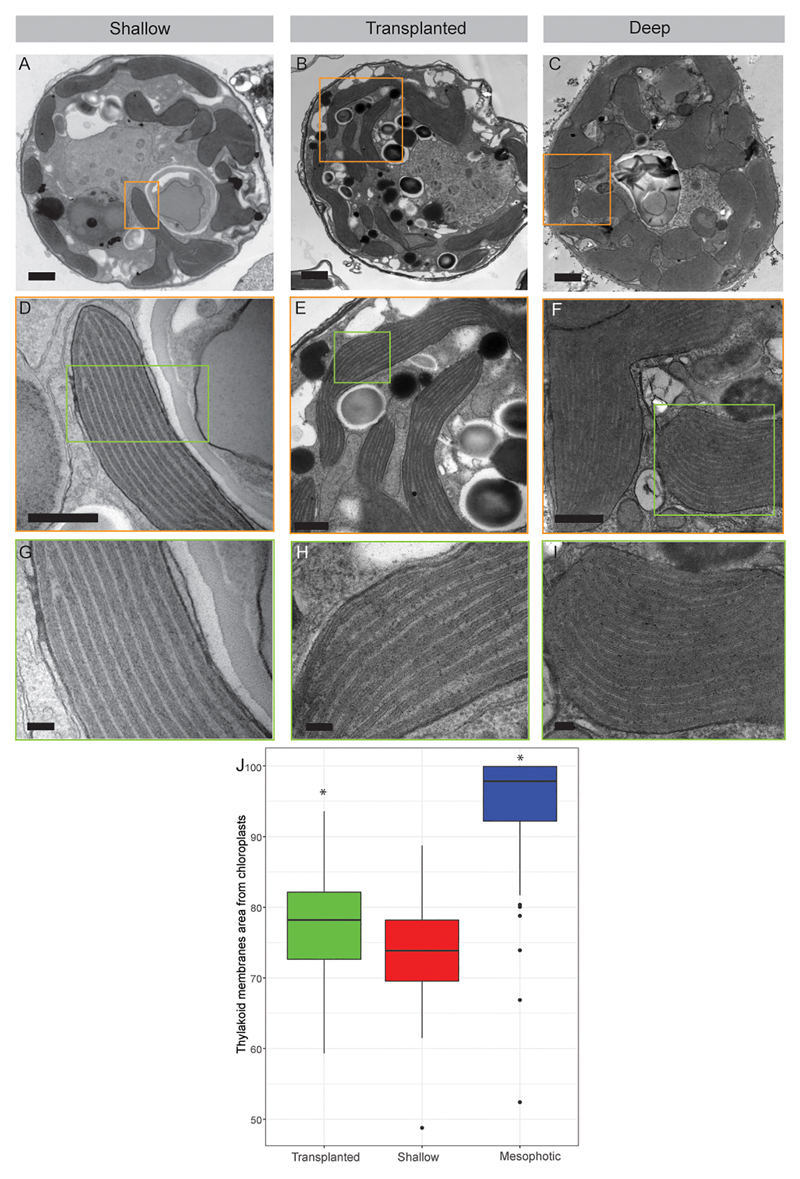
Transmission electron microscopy of symbiont cells. (**A-C**) Whole cell view of the symbiont cells from shallow, transplanted and mesophotic colonies. (**D-F**) High magnification of the chloroplasts delimited by the orange box in (**A-C**). (**G-I**) High magnification of the thylakoid membranes view delimited by the green box in (**D-F**). (**J**) The thylakoid membranes area from chloroplasts is 74 ± 7% in shallow corals, 78 ± 7% in transplanted corals and 95 ± 8% in mesophotic corals. Scale bars: 1 μm (**A-C**), 500 nm (**D-F**), 100 nm (**G-I**). Asterisks (*) indicate statistical differences relative to shallow colonies.

The internal structure of the symbionts, extracted from all three coral groups, was examined using transmission electron microscopy (TEM, Figure 3). Prominent differences were observed in both the volume of the chloroplasts within the cell and the thylakoid membrane density within the chloroplasts. In cells extracted from shallow and transplanted corals, the chloroplasts constitute a smaller percentage of the cell’s volume, compared to cells in the mesophotic colonies. Significant differences were found in the percentage of the photosynthetic area the membranes occupy within the chloroplasts (Figure 3J) between shallow and transplanted (Mann-Whitney, *p* = 0.01, *n* = 70), and between mesophotic and transplanted corals (Mann-Whitney, *p* < 0.0001, *n* = 70). Unfortunately, it is not possible to classify to which species each cell or to morphologically cluster them into two different groups based on the images.

### Trophic Position (TP)

**Figure 4:**
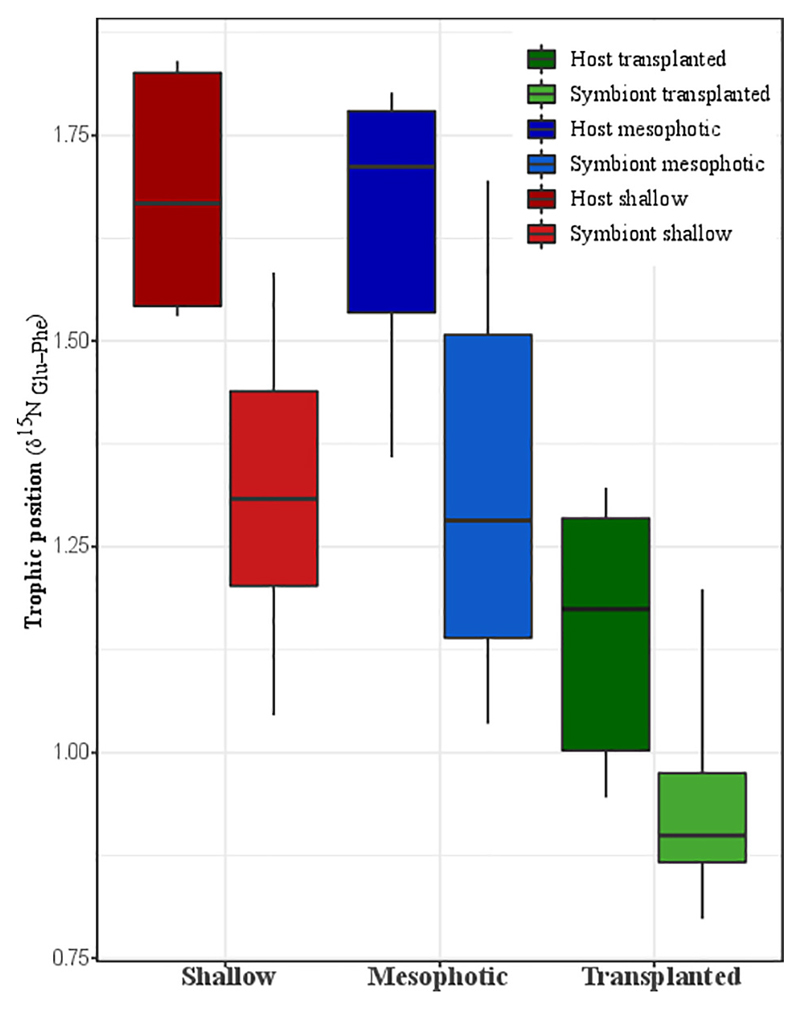
Trophic position calculated trophic position based on nitrogen AA-CSIA of the host and the symbiont from shallow, transplanted, and mesophotic colonies.

The calculated TP from Nitrogen AA-CSIA (glu-phe) of fragments transplanted to the shallow depth demonstrates lower TP than the mesophotic and shallow corals (Figure 4). Both the host and symbiont in the shallow and mesophotic colonies had the same TP of 1.7 ± 0.2 and 1.3 ± 0.2, respectively, while the TP of the host and symbionts of the transplanted fragments was significantly lower with TP of 1.2 ± 0.2 and 0.9 ± 0.2, respectively (PERMANOVA, BH *p* = 0.045).

### Carbon Source

**Figure 5:**
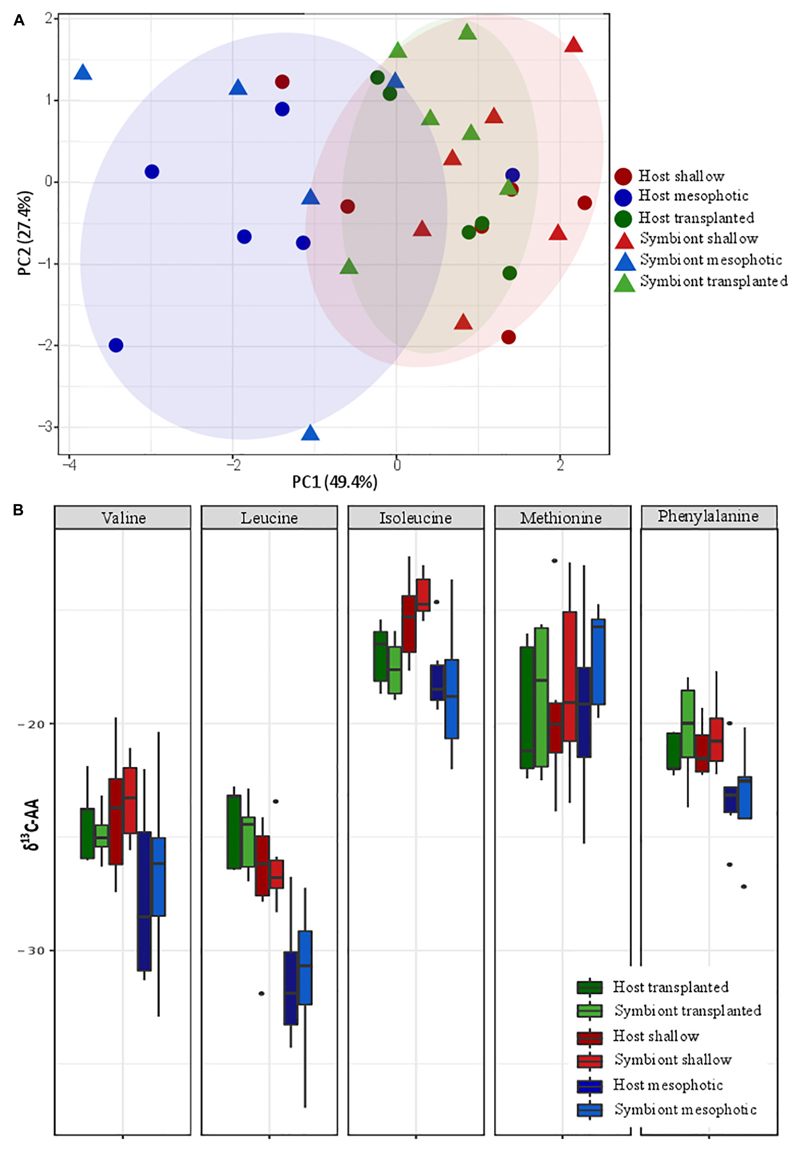
Carbon source (**A**) carbon AA-CSIA PCA of five essential amino acids (valine, leucine, isoleucine, methionine, and phenylalanine). (**B**) Comparison of the different groups AA-CSIA results within each amino acid.

Principal component analysis (PCA) of the δ^13^C of five essential AAs (valine, leucine, isoleucine, methionine, and phenylalanine) of *S. pistillata* host and Symbiodiniaceae samples from the different depth environments show a significant clustering of the transplanted plus shallow fragments versus mesophotic ones (Figure 5A). However, within each group no clustering of host versus symbiont samples is apparent. We further tested the pairwise differences between mesophotic, shallow, and transplanted fragments and found that mesophotic fragments were significantly different than the shallow and the transplanted ones (PERMANOVA, BH *p <* 0.05), suggesting similar carbon sources for these two treatments which differ from the mesophotic colonies. To further evaluate the suggested different carbon sources we examined the δ^13^C of each AA individually (Figure 5B). For all AAs except methionine, the mesophotic colonies exhibited lighter δ^13^C values than shallow colonies or transplanted fragments, but it was only significant for leucine and phenylalanine (PERMANOVA, BH *p <* 0.02). For isoleucine, only the mesophotic and the shallow colonies exhibited a significant difference (PERMANOVA, BH *p* = 0.003).

## Discussion

The energy sources of hermatypic corals, and particularly the balance between autotrophy and heterotrophy at different depths, is an intriguing subject that has not been fully solved. It is especially of interest with regard to depth-generalist corals which occupy a broad depth gradient, such as *S. pistillata* (Loya, 1976). In this study, we discuss the changes with depth regarding the physiological traits in *S. pistillata* and its dinoflagellate symbionts from shallow and mesophotic reefs, particularly in association with different symbiont assemblages, photosynthesis properties, trophic position and carbon sources. By conducting long-term *in situ* transplantation of *S. pistillata* fragments from 60 to 5 m depth, we were able to elucidate the role of predation and photosynthesis in these two habitats. Owing to the fact that the Symbiodiniaceae genera shift in the transplants was very limited, we could distinguish between Symbiodiniaceae-related differences and changes in photo-acclimation. The responses reported here suggest that the acclimation process is both more complex and more limited than previously described (Cohen and Dubinsky, 2015), and might be due to adaptive processes as even after 18 months the transplanted fragments differed in a variety of ways from colonies of the same species from the shallow reef.

Heterotrophy level is commonly calculated based on nitrogen bulk stable isotope analysis (Muscatine et al., 1989; Muscatine and Kaplan, 1994; Einbinder et al., 2009; Houlbrèque and Ferrier-Pagès, 2009; Lesser et al., 2009; Fox et al., 2019; Wall et al., 2020). However, this method is limited to relative trophic position and does not provide absolute values, since it is hard to constrain the isotopic ratio to a baseline (Popp et al., 2007). In this paper we used AA-CSIA to compare glutamic acid and phenylalanine δ^15^N, the two most commonly used amino acids, for calculation of trophic position (Chikaraishi et al., 2009). Interestingly, we found that in contrast to the dogma that corals in low light environments will switch to their alternate source of energy, predation (Muscatine et al., 1989), the coral *S. pistillata* and its associated symbionts collected at shallow and mesophotic reefs have the same TP (host = 1.7 ± 0.2, symbiont = 1.3 ± 0.2) (Figure 4). This means that predation represents the same portion of their total energy consumption. A comparison of host and symbiont TP in the different groups suggests that the host has a higher TP position than the symbiont. The elevated TP in the Symbiodiniaceae, which is expected to have a TP of one, might be explained by the transfer of AAs directly from the host to the symbiont, similar to that observed in *Chlorella* living in symbiosis with the green Hydra (McAuley, 1987). To better understand the TP consequences to the coral nutrition and ecology, we calculated the mass balance and the heterotrophy portion in the diet. The calculation is based on Grossowicz et al. (2020), who calculated a TP of 2.86 using the same method for a heterotroph coral in the Red Sea. We calculated that in order to reach a TP of 1.7 in the host, the mass balance of the heterotrophy proportion should be ~35% of the diet (assuming predation TP is 2.86 and photosynthesis is 1) in both shallow and mesophotic corals. This result is in agreement with the statement that heterotrophy in a healthy coral represents 15-35% of its daily metabolic demand (Houlbrèque and Ferrier-Pagès, 2009).

To better understand the difference between shallow and mesophotic corals’ energy sources, we tested their carbon isotopic source through AA-CSIA of five essential AA (valine, leucine, isoleucine, methionine, and phenylalanine). The δ^13^C AA-CSIA has been used before in corals to trace shifts of carbon sources in paleoclimate (McMahon et al., 2015) and different prey similarities in coral (Grossowicz et al., 2020). Our PCA ordination grouped the shallow corals with the translocated fragments, both of which are well separated from the mesophotic ones (Figure 5A), highlighting a significant difference between them. However, there is no significant difference between the host and the symbiont inside each group, implying that they are sharing the same carbon source or having some kind of carbon cycling between them (Einbinder et al., 2009). Fox et al. (2019) suggested that the differences in carbon sources between different locations originate from variation in predation rate. However, based on nitrogen AA-CSIA we do not find any difference in the TP between them. Moreover, the translocated fragments, which have lower TP, are still grouping with the shallow colonies in the PCA and do not form a separate group, meaning predation is not representing a significant portion of their carbon source or that due to the carbon cycling the essential amino-acids originate from the symbiont regardless of predation. It seems that the existing assumption, according to which the differences in carbon isotope fractionation between mesophotic and shallow corals are due to different symbiont genera (Ezzat et al., 2017; Wall et al., 2020), in our case, is not applicable. That is since our translocated fragments have changed less than 20% of their symbionts to *S. microadriaticum* (inhabiting shallow corals) and retained *C. goreaui* (inhabiting mesophotic corals). AA-specific examination of the carbon isotopic ratio (Figure 5B) reveals that, as previous papers found in carbon bulk analysis, the shallow corals have less negative values (heavier) than the mesophotic ones (Muscatine et al., 1989; Einbinder et al., 2009). Heavier carbon source can suggest a faster photosynthesis rate that is less discriminating between the isotopes (Muscatine et al., 1989; Reynaud-Vaganay et al., 2001; Omata et al., 2008), which is supported by both our observations (Figure 2A) and previous works showing that shallow corals experiencing higher irradiance exhibit elevated photosynthetic rates (Mass et al., 2007; Cohen and Dubinsky, 2015). While the percentage of the total energy sources represented by predation in *S. pistillata* is the same in shallow and mesophotic reefs (according to the TP, Figure 4), the total overall energy consumption in the shallow habitat is higher due to the fact that the photosynthetic rates are higher (deduced from the heavier carbon, Figure 5B). Meaning that predation rates are higher in the shallow reefs. This is in agreement with the higher growth rates that are indicated by the thicker tissue (Figure 1D) who are as well indicative of higher predation rates, since higher predation and photosynthesis rates are correlated with thicker tissue (Ferrier-Pagès et al., 2003; Houlbrèque et al., 2003).

Hermatypic corals and their associated symbiotic algae have at their disposal an assortment of known adaptation mechanisms to depth-dependent changes in light, such as a change in the number and/or type of symbionts, as well as the symbionts’ internal structure, and differences in photosynthetic performance (Chang et al., 1983; Kaiser et al., 1993; Kahng et al., 2019). Indeed, we found significant differences in the algal symbionts’ physiological characteristics among shallow and mesophotic colonies. Fragments from 5 meters showed a higher chlorophyll *a* and symbiont cell surface density compared to deep fragments (Figure 1E), as reflected by the differences in color observed by eye (Figures 1A,B). Moreover, we observed noticeable changes in the symbionts’ photosynthetic performance at different depths, indicative of different adaptive strategies employed by corals living in varying light environments. In fact, mesophotic fragments exhibited a higher photosynthetic efficiency under low light conditions compared to the shallow ones, as indicated by the higher *F_v_/F_m_* (Figure 1C) and the higher YII (Figure 2A). In contrast, fragments from 5 meters showed a better photosynthetic performance and a greater capacity to cope with excess light energy under higher light intensities (Figures 2A,B). In addition, red-shifted chlorophyll fluorescence in shallow corals (Figure 2D) indicates a lower ratio of antennae to reaction centers (Jennings et al., 1993). In deeper water, where light is more limited, this ratio is higher to maximize the absorption of photons. Taken together, these observations indicate that changes in light intensity with depth shape the photosynthetic efficiency and activity of the algal symbionts at multiple levels, as previously observed in *S. pistillata* and other coral species living along broad depth distribution in the Red Sea (Einbinder et al., 2016). This great photosynthetic flexibility among depths was also displayed at the cellular structural level. In shallow corals, the volume of chloroplasts and the density of thylakoid membranes inside the chloroplasts were lower compared to mesophotic fragments (Figure 3), pointing to a reduced number ofphotosynthetic units with decreased depth.

By firstly identifying patterns of energy source, physiological and photosynthetic traits naturally occurring at different light intensities, we were able to determine the acclimation capacity of both the host and the algal endosymbionts in corals transplanted at different depths. In our experiment, the transplanted corals increased the number of Symbiodiniaceae cells (Figure 1F), while the chlorophyll *a* concentrations per total protein decreased nearly to the same levels of shallow colonies (Figure 1H). Transplantation experiments that were done on *S. pistillata* and other species in a natural environment (Winters et al., 2009; Cohen and Dubinsky, 2015; Ben-Zvi et al., 2020) observed the same trend we see here, probably as a result of the higher plankton availability and nutrient sources in the shallow reef. An increase in zooplankton indeed leads to higher symbiont cells density in corals (Muscatine et al., 1989). In contrast, their total protein concentration, though increased, remained more similar to that of the mesophotic colonies (Figure 1D). It was reported that reduced total protein concentration is found in starved corals and/or in corals that cannot have a sufficient budget of heterotrophy (Ferrier-Pagès et al., 2003; Houlbrèque et al., 2003). The transplants also preserved some photosynthetic traits of mesophotic colonies (e.g., the *F_v_/F_m_* values and NPQ, Figures 1C, 2B), demonstrating that the symbiont *C. goreaui* did not completely shift it’s base physiological traits and only partially acclimated to the new conditions. Nevertheless, the symbionts of the transplanted fragments exhibited surprising flexibility and underwent incredible changes attributed to photo-acclimation processes. Further, the symbionts’ fluorescence peak in the shallow and in the transplanted fragments was similar (Figure 2D), meaning that the antennae to reaction centers ratio of the transplants has become similar to that of the shallow colonies. It was recently discovered, in marine cyanobacteria, that enhancement of photosynthetic efficiency could be achieved by tuning the coupling within the antennae complexes (Kolodny et al., 2020), a mechanism that might also be utilized in Symbiodiniaceae and could explain the observed differences in YII (Figure 2A).

The cellular chlorophyll *a* content in the transplants decreased to the same level of shallow colonies (Figure 1I) and, as seen in the TEM analysis, they reduced the number of photosynthetic units by (1) decreasing the volume of their chloroplasts and (2) reducing the density of thylakoid membranes inside the chloroplasts (Figure 3). Through both mechanisms, they now resemble the characteristics of the shallow corals’ symbiont cells. Although it was reported that most coral species do not switch their symbiont types in response to environmental changes, corals species that harbor several symbiont species, such as *S. pistillata,* are more susceptible to switching their symbionts (Goulet, 2006). The stability of the coral-algal mutualism in *S. pistillata* over long-term acclimation was not determined until today. In our study, only 20% of the shallow-transplants’ symbionts belonged to the shallow Symbiodiniaceae genus *(S. microadriaticum)*. Therefore, we can conclude that the observed differences are a result of acclimation of *C. goreaui* rather than simply an exchange of genera. Such photo-acclimation mechanisms were shown in Symbiodiniaceae cultures grown under different light intensities (Lesser and Shick, 1990) and from corals acclimated to low and high light (Falkowski and Dubinsky, 1981; Iluz and Dubinsky, 2015).

In terms of photo-protective mechanisms, the transplanted corals did not develop an NPQ mechanism to handle the excess light of their new shallow environment, as evidenced by their NPQ levels that remained as low as that of the mesophotic colonies (Figure 2B). In a previous study (Einbinder et al., 2016), *S. pistillata* corals transplanted from 3 to 65 m at the same study site, showed an exact mirror image of our results. The fragments transplanted from shallow to mesophotic depths maintained their original Symbiodiniaceae species (S. *microadriaticum)* and their NPQ traits remained similar to that of shallow colonies, indicating that they did not acclimate to their new light regime in that sense. The fact that these two genera of Symbiodiniaceae, both in the current study and the one reported by Einbinder et al. (2016), did not shift their NPQ traits in response to the light level and spectrum, clearly indicates that NPQ of the symbiont is an adaptive process. Although the exhibited NPQ did not improve, increased reliance on PSII repair mechanisms was shown in Symbiodiniaceae cells of *S. pistillata* that were acclimated to high light (Jeans et al., 2013), indicating that while photoacclimation is not evident in their NPQ mechanism, they do undergo other changes to cope with the higher light intensity. Furthermore, the host plays an important role here in potentially augmenting auxiliary processes by upregulation of mitochondria, oxidative-stress, oxidoreductase and response to UV gene ontological categories of the transplanted fragments, as reported by Malik et al. (2020).

Our results show that transplanted corals from mesophotic to shallow reef exhibited extensive but not complete acclimation to shallow conditions. The photosynthetic apparatus of the transplanted fragments is quite acclimated to the shallow water, and consequently, the transplants show carbon isotopic signatures of heavier carbon compared to mesophotic corals (Figure 5), resembling the shallow corals in this regard. However, the trophic position of the transplanted corals (host =1.2 ± 0.2, symbiont = 0.9 ± 0.2) is significantly decreased compared to both mesophotic and shallow corals (Figure 4), implying that their heterotrophic ability did not improve substantially. Therefore, heterotrophy represents a smaller portion. We calculated the mass balance to be only ∼10% of their diet. This suggests that the energy budget of transplants is more dependent on photosynthesis compared to corals from shallow habitat. Malik et al. (2020) showed at the same study site that even after 15 months *S. pistillata* colonies that were transplanted from 60 to 5 m depth still did not gain the same polyp morphology as the shallow corals, remaining more similar to mesophotic corals. This may point that the host is not fully acclimated and therefore is less capable of predation than local shallow corals.

## Conclusion

Based on the comparison between the natural shallow and mesophotic colonies, relying on the transplants as a control experiment, we conclude that heterotrophy in the mixotrophic coral has a major role regardless of the depth and light condition, representing ~35% of the nutritional source. Hence, in our work, the ratio between heterotrophy and autotrophy is the same in the well-lighted environment of the shallow water and the lower-lighted mesophotic zone but the total energy acquisition is higher in the shallow reef. Therefore, to maintain the predation photosynthesis proportion, shallow corals have a larger pray capacity than the mesophotic ones. In both zones, the main source for carbon is from the photosynthesis pathway and the difference in carbon isotopic ratio is due to different photosynthetic carbon assimilation rates (Muscatine et al., 1989; Reynaud-Vaganay et al., 2001; Omata et al., 2008) and not predation or different Symbiodiniaceae.

In corals transplanted from mesophotic to shallow habitats, we observed several different mechanisms of photo-acclimation: algae coverage expanded to all sides of the fragment; limited exchange of symbionts genera from *C. goreaui* to *S. microadriaticum,* of 20% after 18 months; increased cellular chlorophyll *a* in the symbionts which result from the decreased volume of chloroplasts and decreased density of thylakoid membranes within the chloroplasts; and lower antennae to photosystems ratio. However, some traits, such as NPQ, are adaptive characteristics of the specific Symbiodiniaceae species, which does not change in response to the high light regime in the shallow reef. Moreover, the acclimation seen in the host itself is limited as evidenced by total protein concentration, and skeleton morphology (Malik et al., 2020) which remain more similar to the mesophotic corals even after 18 and 15 months, respectively. While transplants increased their photosynthetic rates to match those of shallow reef corals, their predation rates remained lower than those of shallow reef corals. Our results suggest that while mesophotic reefs could serve as a potential refuge source for shallow reefs, the transition is complex, as even at time scales of 18 months the acclimation is only partial.

## Supplementary Material

The Supplementary Material for this article can be found online at: https://www.frontiersin.org/articles/10.3389/fmars.2020.566663/full#supplementary-material

Supplementary material

## Data Availability

The original contributions presented in the study are included in the article/Supplementary Material, further inquiries can be directed to the corresponding author.

## Footnotes

1
http://www.ncbi.nlm.nih.gov/BLAST
