# Supplementary material for: Energy Sources of the Depth-Generalist Mixotrophic Coral Stylophora pistillata
Source: Front Mar Sci. Author manuscript; Available in PMC 2021 Jan 5. (PMC7116548; doi:10.3389/fmars.2020.566663)
Supplement: Supplementary material [file EMS108452-supplement-Supplementary_material.DOCX]

Supplementary Material

Energy sources of the depth-generalist mixotrophic coral *Stylophorapistillata*

Stephane Martinez^1, 2#^, Yuval Kolodny^3, 4#^, Eli Shemesh^1^, Federica Scucchia^1, 5^ ,Reinat Nevo^6^, Smadar Levin‐Zaidman^7^, Yossi Paltiel^3, 4^, Nir Keren^8^,Dan Tchernov^1, 2^, Tali Mass^1, 2*^

^1^Department of Marine Biology, The Leon H. Charney School of Marine Sciences, University of Haifa, Mt. Carmel, Haifa 3498838, Israel

^2^Morris Kahn Marine Research Station, The Leon H. Charney School of Marine Sciences, University of Haifa, Sdot Yam, Israel

^3^Applied Physics Department, The Hebrew University of Jerusalem, Jerusalem 91904, Israel

^4^The Center for Nanoscience and Nanotechnology, The Hebrew University of Jerusalem, Jerusalem 91904, Israel

^5^TheInteruniversity Institute of Marine Sciences, Eilat 88103, Israel

^6^Department of Biomolecular Sciences, Weizmann Institute of Science, Rehovot, Israel

^7^Department of Chemical Research Support, Weizmann Institute of Science, Rehovot, Israel

^8^Department of Plant and Environmental Sciences, The Alexander Silberman Institute of Life Sciences, The Hebrew University of Jerusalem, Jerusalem 91904, Israel

# These authors contributed equally to the work

*** Correspondence:**Tali Masstmass@univ.haifa.ac.il

Supplementary table 1: *Stylophora pistillata* physiological parameters: specific values of total protein, chlorophyll a and symbiont cells per surface area; number of symbiont cells and chlorophyll a per total protein; cellular chlorophyll a concentration in the symbiont cells; and maximal photosystem II quantum yield ($F_{v}/F_{m}$) obtained by Imaging-PAM

Supplementary table 2: *Stylophora pistillata* specific amino acids δ^13^C values of corals from shallow reef (5m), mesophotic reef (60m) and transplanted fragments from 60m to 5m. From each fragment, host and symbiont are analyzed separately. Isotopic data are corrected by removing the added carbons from the derivatization process (see material and methods).

Supplementary table 3: *Stylophora pistillata* specific amino acids δ^15^N values and trophic position (TP) of corals from shallow reef (5m), mesophotic reef (60m) and transplanted fragments from 60m to 5m. From each fragment, host and symbiont are analyzed separately. The trophic position was calculated from the equation $\text{TP}\text{(Glu/}\text{Phe}\text{)}\text{=((δ}\text{15}\text{N}\text{Glu}\text{–δ}\text{15}\text{N}\text{Phe}\text{–β)/TDF}\text{AA}\text{)+1 }$(Chikaraishi et al., 2009) where β=-0.36 and TDF_AA_=4.54 (Martinez et al., 2020).
